# Genetic Evidence for Metabolites Mediated Causal Inference Between Gut Microbiota and Venous Thromboembolism

**DOI:** 10.3390/genes17091098

**Published:** 2026-09-11

**Authors:** Pin Huang, Wenxiao Wei, Ying Xiao, Qian Zhu

**Affiliations:** 1 Department of Intensive Care Unit (ICU), Sun Yat-sen University Cancer Center, Guangzhou 510060, China; huangpin@sysucc.org.cn (P.H.); weiwx@sysucc.org.cn (W.W.); 2 State Key Laboratory of Oncology in South China, Guangdong Key Laboratory of Nasopharyngeal Carcinoma Diagnosis and Therapy, Guangdong Provincial Clinical Research Center for Cancer, Sun Yat-sen University Cancer Center, Guangzhou 510060, China

**Keywords:** gut microbiota, metabolite, venous thromboembolism, mendelian randomization

## Abstract

**Background**: More efficient and safe preventive treatments for venous thromboembolism (VTE) are warranted to decrease the incidence of hemorrhage. Emerging evidence has highlighted the pivotal role of the intestinal microbiota alterations and their metabolites in the pathophysiology of VTE process. However, the specific species involved the VTE process and the potential underlying mechanisms remain unclear. **Methods**: The causal relationships between 82 species and VTE were assessed using two-sample Mendelian Randomization (MR) analyses. Instrumental variables (F > 10) without horizontal pleiotropy were selected using the PhenoScanner and MR PRESSO global test. Subsequently, causal estimates were primarily derived using the inverse variance weighted method. The reliability of the results was further examined utilizing various sensitivity analyses, reverse MR analysis and multivariate MR analysis. Additionally, the potential mediated effects of gut microbiota-related metabolites were explored and replicated using the mediation MR analysis. Moreover, genome annotation and pathway enrichment analysis were conducted to investigate the role of *CAG-81 sp000435795* in the related metabolites metabolism. **Results**: We identified independent and positive causal effects of *CAG-81 sp000435795* and *CAG-877 sp000433455* on VTE, which remained robust across multiple sensitivity analyses. Mediation analysis further revealed that the causal effect of *CAG-81 sp000435795* on VTE risk was partially mediated by albumin, Omega-3 fatty acids and polyunsaturated fatty acids (PUFA), which were all validated in the replicated cohorts. Furthermore, *CAG-81 sp000435795* might participate in the metabolism of albumin and fatty acids. **Conclusions**: Our findings provide evidence supporting a causal role of *CAG-81 sp000435795* in the VTE pathogenesis and implicate microbes associated metabolites, albumin and Omega-3, as potential mediators.

## 1. Introduction

Venous thromboembolism (VTE) not only causes substantial morbidity and mortality but also incurs high healthcare expenditures due to prolonged treatment and potential complications [1]. For now, anticoagulant therapy and mechanical prophylaxis are the primary preventive treatments for VTE. Nevertheless, bleeding risks remain unresolved in VTE prevention [2], especially in patients with critical illness [3]. And mechanical prophylaxis for VTE is inefficient [4,5]. Furthermore, a considerable number of patients still experience recurrent events [6], highlighting the urgent need to elucidate novel pathogenic mechanisms and identify more precise therapeutic targets.

In recent years, emerging evidence has highlighted the critical role of the intestinal microbiota alterations in the VTE process [7,8]. However, conventional observational studies are often limited by potential confounding factors and reverse causality, which hinder the identification of true causal relationships. The utilization of instrumental variables (IVs) with genetic variants, which are randomly allocated based on random Mendelian genetic variation, Mendelian randomization (MR) has been widely applied to explore the causal role of exposure on outcome risk to minimize the influence of confounding factors and the research costs [9]. Several studies have demonstrated potential causal relationships between gut microbiota and VTE at the genus level with MR analysis [10,11,12]. However, the associations between specific species and VTE are still unclear.

The gut microbiota participates in multiple physiological processes, including digestion, immune regulation, and energy metabolism. The gut microbiota through metabolic activities generate a variety of metabolites, including short-chain fatty acids, bile acids, trimethylamine N-oxide (TMAO), and amino acid derivatives [13]. For instance, TMAO, derived from the gut microbiota-mediated metabolism of dietary phosphatidylcholine and choline, has been firmly associated with cardiovascular diseases by promoting endothelial dysfunction, platelet activation, and thrombosis. Moreover, short-chain fatty acids play vital roles in modulating immune responses and maintaining vascular integrity [14]. What is more, the translocation of lipopolysaccharide from Gram-negative bacteria into the systemic circulation results in endotoxemia [15]. And study has shown that elevated plasma lipopolysaccharide-binding protein was associated with a higher VTE risk in women [16]. These findings suggest that metabolites may serve as key mediators through which the gut microbiota influences the development of VTE. However, the precise mechanisms by which metabolites bridge the gut microbiota and VTE remain largely elusive. Understanding the intricate interplay among the gut microbiota, its metabolites, and VTE could potentially unveil new preventive and therapeutic strategies.

Thus, by leveraging Mendelian randomization analysis based on large-scale genome-wide association studies (GWAS), the present study aims to provide robust evidence for a causal role of specific gut microbial species in influencing VTE risk. Then we aim to comprehensively explore the metabolites-mediated association between the gut microbiota and venous thrombosis. It will be helpful for providing novel insights into VTE pathogenesis and facilitating the development of innovative treatment approaches.

## 2. Methods

### 2.1. Data Sources

#### 2.1.1. Gut Microbiota Cohorts

The 209 species summary data from 5,959 individuals were extracted from the FINRISK 2002 study, including men and women aged between 25 and 74 years from six geographical areas of Finland [17].

As the original study showed, the genotyping was performed with Illumina genome-wide SNP arrays, and the associations between the relative abundance of taxa and genetics were calculated with a linear mixed model adjusting for the top ten genetic principal components, age, sex, and genotyping batch.

The GWAS data were extracted from the MRC Integrative Epidemiology Unit (IEU) open GWAS project (https://openGWAS.io/datasets/ (accessed on 19 May 2025)) [18,19].

#### 2.1.2. VTE Cohorts

VTE genetic data were from the most extensive VTE GWAS meta-analysis to date, encompassing six cohorts of 81,190 European cases with VTE and 1,419,671 European controls. The diagnosis of VTE was made using the International Classification of Diseases, Tenth Revision (ICD-10) coding system. And the detailed codes were available in the Supplementary Note of Ghouse J et al. [20].

The measurement, quality control and selection of genetic variants were described in the original meta-analysis supplemental materials. The summary data were obtained from https://www.decode.com/summarydata (accessed on 31 Augst 2023).

#### 2.1.3. Metabolites Cohorts

Firstly, we explored 172 plasma biomarkers of lipoprotein lipids, fatty acids, and small molecules such as amino acids, ketones, and glycolysis metabolites using plasma nuclear magnetic resonance (NMR) methods for 118,461 participants in the UK Biobank [21]. The GWAS data were extracted from the MRC IEU open GWAS project (https://openGWAS.io/datasets/ (accessed on 6 June 2025)) [18,19].

Lipopolysaccharide data (*n* = 11,296) from Leskelä J’s study [15] (ID: ebi-a-GCST90032674), and lipopolysaccharide-binding protein dataset (*n* = 10,708) from Pietzner M’s study [22] (ID: ebi-a-GCST90019429) were extracted from MRC IEU open GWAS project (https://openGWAS.io/datasets/ (accessed on 6 June 2025)) [18,19].

Three additional GWAS datasets were used for replication. The albumin GWAS data from Mbatchou J (*n* = 407,746) [23] (ID: ebi-a-GCST90013990) were obtained from MRC IEU open GWAS project (https://openGWAS.io/datasets/ (accessed on 19 November 2025)). The polyunsaturated fatty acid (PUFA) (ID: GCST90302071) and the Omega-3 fatty acid (ID: GCST90301959) GWAS data from Karjalainen MK (*n* = 136,016) [24] were obtained from the GWAS Catalog database (https://www.ebi.ac.uk/gwas/ (accessed on 19 November 2025)).

#### 2.1.4. Ethical Principles

This study was based on publicly available summary data. The original study protocols previously mentioned were all approved by the local Ethical Committee. Thus, no extra ethical approvals were needed.

The comprehensive description of datasets in the study were described in the Supplemental Table S1.

### 2.2. Study Design

**Figure 1:**
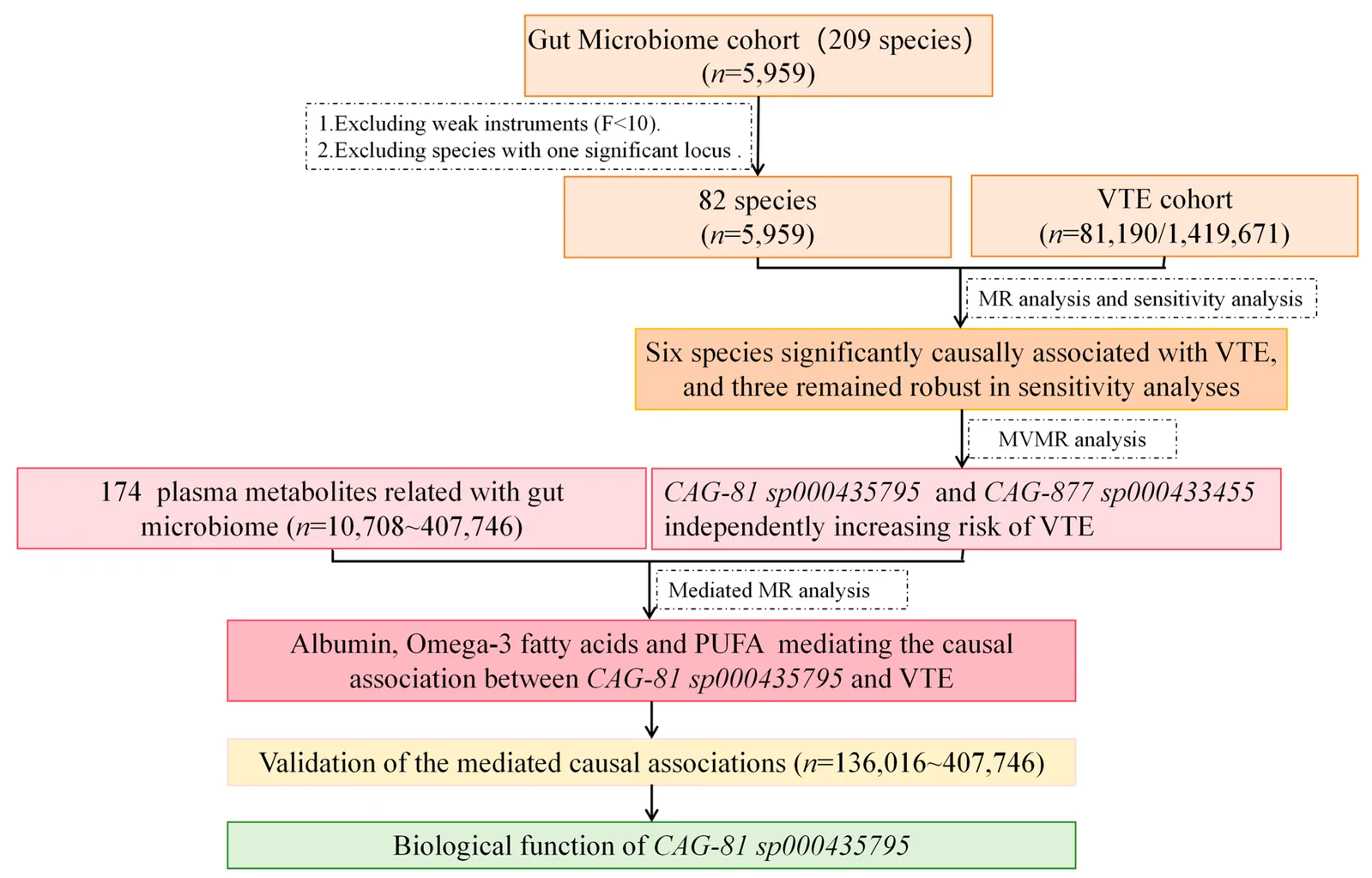
Overview of MR Analysis. VTE: venous thromboembolism; MR: Mendelian Randomization analysis; MVMR: multivariate Mendelian Randomization analysis; PUFA: polyunsaturated fatty acid.

The overview of the study was depicted in Figure 1. Additionally, the study was guided by STROBE-MR [25] (Supplemental Table S2).

### 2.3. Instrumental Variables Selections

Firstly, the instrumental variables of gut microbiota were extracted in accordance with the below criteria, also satisfying the instrumental variables (IVs) necessary assumptions at the MR study [26]:

i. The selection of single nucleotide polymorphisms (SNPs) (*p* < 1.0 × 10^−5^) associated with each species as potential IVs was conducted. According to the previous studies, the threshold of *p* < 1.0 × 10^−5^ exhibited the largest explained variance on microbial features [27].
ii. The present study examined the linkage disequilibrium (LD) between IVs with reference to the 1000 Genomes European Project. The inclusion criteria for SNPs were r^2^ < 0.01 and window size > 10,000 kb to avoid the influence of LD between IVs.
iii. In order to enhance the reliability of causal estimates, SNPs with intermediate allele frequencies were excluded from the analysis. These SNPs have the potential to lead to non-directional or attenuated instrument effects.
iv. IVs with strong associations (*p* < 5.0 × 10^−8^) with either VTE or established confounders were excluded to minimize the potential bias resulting from horizontal pleiotropy. In light of smoking’s established role as a risk factor for VTE, SNPs associated with smoking behavior were identified using the PhenoScanner [28] and removed from the instrument set. We further applied the MR-PRESSO framework to detect and address potential pleiotropic outliers. Specifically, the MR-PRESSO global test and distortion test (both at *p* < 0.05) were used to assess the presence of significant horizontal pleiotropy. When either test indicated pleiotropy, an outlier-corrected MR analysis was performed. If the causal results changed substantially after outlier removal, the outlier SNP(s) were excluded from subsequent analyses.
v. It has been demonstrated that weak IVs in MR analyses can introduce bias due to insufficient strength in predicting exposure. The F-statistic is a common tool employed in the assessment of the validity and strength of genetic instruments, with values below 10 generally indicating weak instrument bias. This statistic was derived from the following formula: $F=(\frac{n-k-1}{k})(\frac{R^{2}}{1-R^{2}})$ [29]. Where *n* denoted the sample size of the exposure dataset, *k* was the number of genetic variants used as instruments, and genetic variants (*R*^2^) represented the proportion of variance in the exposure phenotype explained by these variants.

### 2.4. Statistical Analysis

#### 2.4.1. MR Analysis and Sensitivity Analyses for Gut Microbiome and VTE

SNPs satisfying all the aforementioned criteria were extracted as IVs for subsequent MR analysis. Firstly, the bacterial taxa at the species level by phylum types significantly associated with VTE were verified using two-sample MR analysis. Primary causal estimates were derived using the inverse-variance weighted (IVW) method [30]. False discovery rate (FDR) using the Benjamini–Hochberg procedure was calculated to correct for multiple tests across all tested bacterial species and metabolites. To find more potential signals between gut microbiome and VTE, we adopted an FDR threshold of 0.2 [31]. To triangulate evidence and verify the MR assumptions between bacterial taxa and VTE, several alternative approaches were also carried out, including MR-Egger, weighted median method, weighted mode, simple median method and MR-PRESSO [32,33,34,35].

To evaluate the validity of the MR findings, we conducted a series of sensitivity analyses. Firstly, heterogeneity was quantified using Cochran’s Q statistic [30]. In the absence of heterogeneity (*p* > 0.05), the IVW fixed model would be employed. The analyses carried out with the IVW random effect model while there is heterogeneity (*p* < 0.05). Secondly, scatter plots and funnel plots were used to assess IV heterogeneity [36]. Due to the limited accuracy of the MR Egger regression in specific cases, the horizontal pleiotropy was assessed using the MR-Egger regression and the MR-PRESSO method (global test and distortion test), with *p* < 0.05 considered statistically significant [35,37]. When evidence of the horizontal pleiotropy was detected, we performed outlier-corrected MR analyses after removing the influential SNPs with horizontal pleiotropy. Additionally, to identify the influential outlier variants, we carried out leave-one-out analyses by iteratively removing each SNP and recalculating the causal estimate. Any instrument whose exclusion materially altered the direction or magnitude of the primary IVW estimate was flagged as a potential outlier. To corroborate causal directionality, we conducted bidirectional MR using independent genome-wide significant loci as IVs, complemented by Steiger filtering to test the orientation of causation. Finally, to disentangle direct effects from potential confounding among correlated exposures, we performed multivariable MR (MVMR) using a weighted regression model, conditioning on the significant species identified in the two-sample MR analyses [38].

#### 2.4.2. Mediated MR Analysis

Whether gut microbiome-derived plasma metabolites mediated the causal association between species and VTE was investigated using mediated MR analysis. Using the same methods as those in Section 2.4.1, we first explored the causal associations between plasma metabolites and VTE. Then MR analyses were carried out between species significantly associated with VTE and the plasma metabolites which were verified to significantly affect the risk of VTE in the first step. Reverse MR analyses and the Steiger tests were performed to verify the causal directions. Furthermore, we validated the causal associations in other cohorts.

Furthermore, scatter plots, funnel plots and leave-one-out analyses were used to assess IVs heterogeneity. And to disentangle direct effects from potential confounding among correlated exposures, we performed a joint MVMR analysis, conditioning on the strongly correlated metabolites identified in the mediated MR analyses.

The proportion of mediation by metabolites affecting the causal associations between gut microbiota and VTE was calculated using the indirect effect. And the indirect effect is calculated using the following formula: *b*_12_ = *b*_1_ × *b*_2_. Here, *b*_1_ represents the causal effect between gut microbiota and plasma metabolites and *b*_2_ represents the causal effect between plasma metabolites and VTE. And the mediation proportion uses the following formula: mediation proportion = (indirect effect/total effect) × 100%. The total effect is the causal effect between gut microbiota and VTE. The 95% CI for the mediation effects and proportions mediated were estimated using the delta method [39].

All statistical analyses were performed in R 4.3.1 utilizing the ‘TwoSampleMR’ [40], ‘MVMR’, ‘ggplot2’ and ‘forestploter’ packages.

#### 2.4.3. Function Analysis of *CAG-81 sp000435795*

Annotation of the *CAG-81 sp000435795* genome was performed using Bakta in Proksee [41], followed by annotation utilizing the Rapid Annotation using Subsystem Technology (RAST) server to compare functional categorization and subsystem coverage [42,43].

## 3. Results

### 3.1. Causal Associations Between Gut Microbiome and VTE

#### 3.1.1. Instrumental Variables Selection

With gut microbiota as the exposure variable, Mendelian Randomization analysis on locus-significant SNPs (*p* < 1.0 × 10^−5^) of 209 species was carried out. Excluding weak instruments (F < 10), 104 species remained in the study. 22 species with one locus-significant SNP were also excluded. Overall, 82 species with 1349 instrumental variables (10 < F < 127) were included in the study (detailed SNPs as seen in Supplemental Table S3).

While VTE was the exposure variable, 128 independent, significant SNPs (*p* < 5.0 × 10^−8^) from the VTE GWAS meta-analysis were extracted (F > 10) (detailed SNPs as seen in Supplemental Table S4).

#### 3.1.2. Mendelian Randomization Analysis Results Between Gut Microbiome and VTE

**Figure 2:**
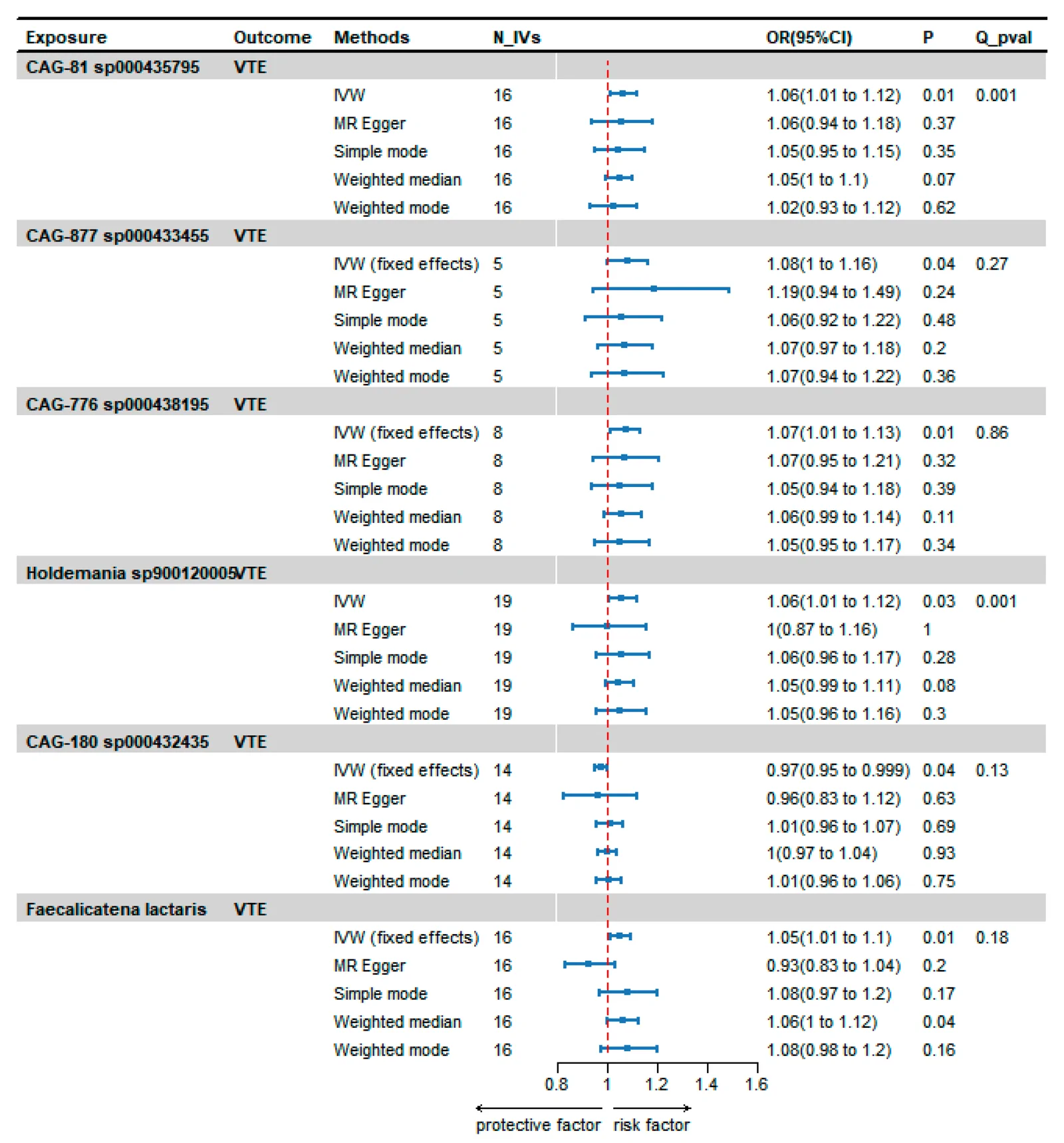
Forest plot of MR results between gut microbiome and VTE. The x-axis shows odds ratios and their 95% confidence intervals. N_IVs: number of instrumental variables; OR: odds ratio; CI: confidence interval; VTE: venous thromboembolism; MR: mendelian randomization analysis; Q_pval: the *p* value of heterogeneity analysis with Cochran’s Q statistic.

MR analyses were first performed to examine the causal associations between 82 species and VTE. All detailed results were presented in Supplemental Table S6. As shown in Figure 2, six species were causally associated with VTE. Among the identified species, five species were suggestively positively associated with VTE risk: *CAG-81 sp000435795* (OR: 1.06; 95% CI: 1.01 to 1.12; *p*: 0.01; FDR: 0.16), *CAG-776 sp000438195* (OR: 1.07; 95% CI: 1.01 to 1.13; *p*: 0.01; FDR: 0.11), *Holdemania sp900120005* (OR: 1.06; 95% CI: 1.01 to 1.12; *p*: 0.03; FDR: 0.15), *CAG-877 sp000433455* (OR: 1.08; 95% CI: 1.00 to 1.16; *p*: 0.04; FDR: 0.15), and *Faecalicatena lactaris* (OR: 1.05; 95% CI: 1.01 to 1.1; *p*: 0.01; FDR: 0.16) Conversely, *CAG-180 sp000432435* was probably associated with a decreased risk of VTE (OR: 0.97; 95% CI: 0.95 to 0.999; *p*: 0.04; FDR: 0.43). The causal directions were further verified by the Steiger test and reverse MR analysis (Supplementary Tables S10 and S11). According to the leave-one-out analyses of each biome species, all of their single SNPs were consistent with the overall MR analysis (Supplemental Figures S1–S6A). Scatter plots showed no obvious outliers (Supplemental Figures S1–S6B). Furthermore, funnel plots for the IVW method showed approximate symmetry of the included instrumental variables for each species (Supplemental Figures S1–S6C).

The results from supplementary MR methods (MR Egger, Simple mode, weighted median, and weighted mode) were consistent with the MR results from IVW methods except *CAG-180 sp000432435* and *F. lactaris*. Therefore, additional evidence is required to verify causal associations between *CAG-180 sp000432435*, *F. lactaris* and VTE.

Among the six species examined, the Egger Intercept test indicated possible horizontal pleiotropy only for *F. lactaris* (*p* = 0.03). However, the MR-PRESSO distortion and outlier test did not identify any outlier SNPs that could lead to significant horizontal pleiotropy (Supplemental Table S9). Consequently, *F. lactaris* was excluded for further MR analysis. Among the six species examined, the MR-PRESSO distortion and outlier test identify outlier SNPs only for *CAG-81 sp000435795* and *Holdemania sp900120005*. After excluding the outlier SNP of *CAG-81 sp000435795*, the MR result remained consistent with those obtained before outlier exclusion. However, after excluding the outlier SNPs for *Holdemania sp900120005*, the causal association was no longer statistically significant (P_Outlier-corrected = 0.07). Thus, *Holdemania sp900120005* was also excluded from the subsequent MR analysis.

#### 3.1.3. Results of Multivariate Mendelian Randomization Analysis

To distinguish the true causal contributions of multiple species to VTE, multivariate Mendelian Randomization analysis was carried out. As shown in Table 1, *CAG-776 sp000438195* was no longer causally associated with VTE (beta: 0.03, *p*: 0.44). *CAG-81 sp000435795* (beta: 0.06, *p*: 0.004) and *CAG-877 sp000433455* (beta: 0.12, *p*: 0.03) dependently increased the risk of VTE.

### 3.2. Metabolites Mediating Associations Between Gut Microbiome and VTE

#### 3.2.1. Causal Relationships Between Metabolites and VTE

**Figure 3:**
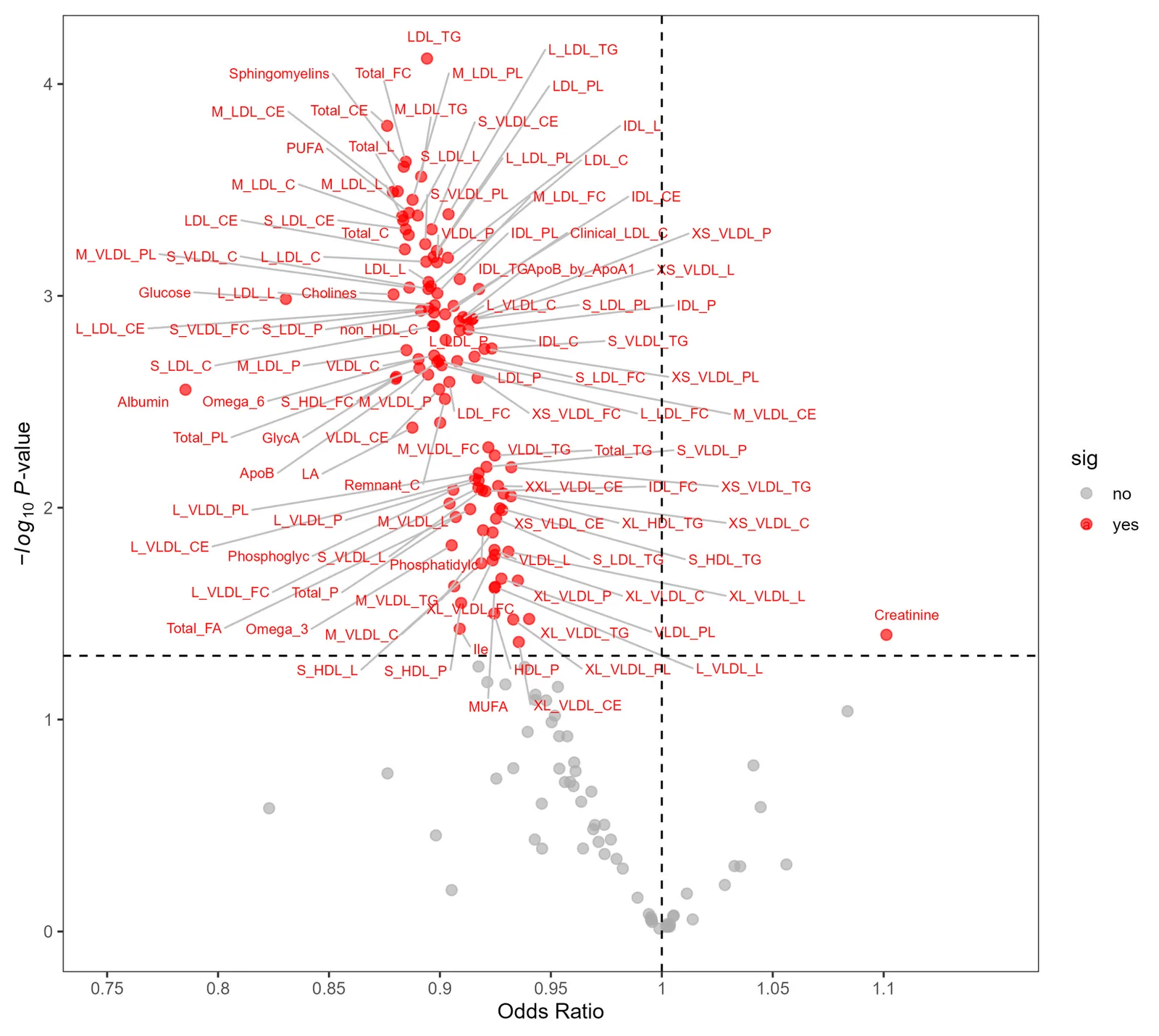
Volcano plot of MR analysis for plasma metabolites on VTE. OR: odds ratio.

The 10,877 IVs (F > 10) of 172 metabolites were included in the MR analysis (Supplemental Table S5). As Figure 3 shown, 103 metabolites significantly decreased the risk of VTE (*p* < 0.05, FDR < 0.05, Supplemental Table S7). Six metabolites (Concentration of HDL particles, Triglycerides in very large VLDL, Phospholipids in very large VLDL, Isoleucine, Cholesteryl esters in very large VLDL and Creatinine) were suggestively associated with VTE. Bidirectional effects among total lipids in small HDL, cholesterol in large VLDL, concentration of small HDL particles, and Cholesteryl esters in very large VLDL on VTE were found with reverse MR analysis (Supplemental Table S11).

Six IVs for LPS and four IVs for LPS binding protein were extracted. No statistically significant evidence of a causal effect between LPS and VTE was observed (OR: 1.07; 95% CI: 0.93 to 1.23; *p*: 0.35; p_het:7.62 × 10^−8^). We did not find the causal association between Lipopolysaccharide-binding protein and VTE (OR: 1.00; 95% CI: 0.99 to 1.02; *p*: 0.79; p_het:0.40). Based on the Egger Intercept test, no significant horizontal pleiotropy was detected in the MR analysis between LPS (p_egger:0.41)/LPS binding protein (p_egger:0.38) and VTE.

#### 3.2.2. Causal Relationships Between Species and Metabolites

To further explore potential mediating pathways, we examined the causal associations of the two species (*CAG-81 sp000435795* and *CAG-877 sp000433455*) and 104 metabolites (the detailed MR results in Supplemental Table S8). Only *CAG-81 sp000435795* showed significant associations with metabolites linked to VTE. Specifically, *CAG-81 sp000435795* was associated with decreased levels of albumin (OR: 0.97; 95% CI: 0.94 to 0.99; *p*: 0.003), Omega-3 fatty acids (OR: 0.94; 95% CI: 0.90 to 0.99; *p*: 0.009) and polyunsaturated fatty acids (OR: 0.97; 95% CI: 0.95 to 0.997; *p*: 0.03). The directionality of these causal effects was confirmed by Steiger filtering test (*p* < 0.001, Supplemental Table S10). Furthermore, reverse MR analysis indicated no evidence of reverse causation from *CAG-81 sp000435795* to the three metabolites (*p* > 0.05, Supplemental Table S11).

To test the robustness of the MR results, The leave-one-out analyses of *CAG-81 sp000435795* on albumin (Supplemental Figure S7A), Omega-3 (Supplemental Figure S8A) and PUFA (Supplemental Figure S9A) showed that after excluding their single SNP the direction was all consistent with the overall MR analysis. Scatter plots showed no obvious outliers (Supplemental Figures S7–S9B). Furthermore, funnel plots for the IVW method showed approximate symmetry of the included instrumental variables for each metabolites (Supplemental Figures S7–S9C).

We also did the sensitivity test for albumin IV, Omega-3 and PUFA IVs. There was no obvious outlier SNP that could affect the MR results from the three metabolites to VTE (Supplemental Figures S10–S12).

**Figure 4:**
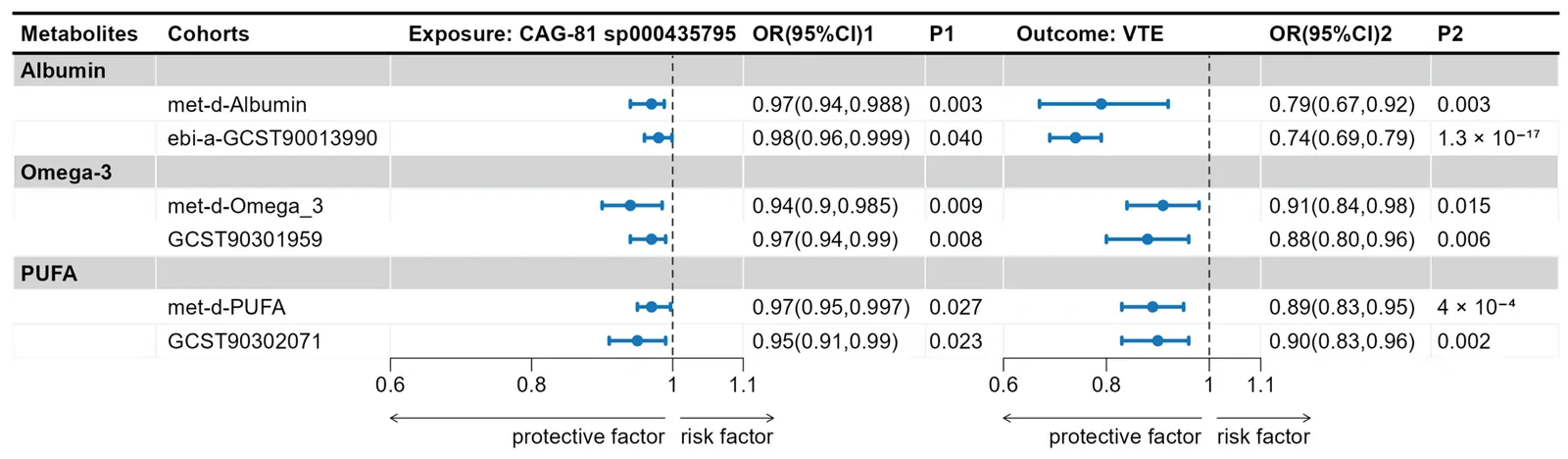
Forest plot of mediation MR analysis. The x-axis shows odds ratios and their 95% confidence intervals. VTE: venous thromboembolism; OR: odds ratio; CI: confidence interval; MR: mendelian randomization analysis; PUFA: polyunsaturated fatty acid.

To validate the results, we also tested the associations in other cohorts. As shown in Figure 4, the validation sets were consistent with our results.

**Figure 5:**
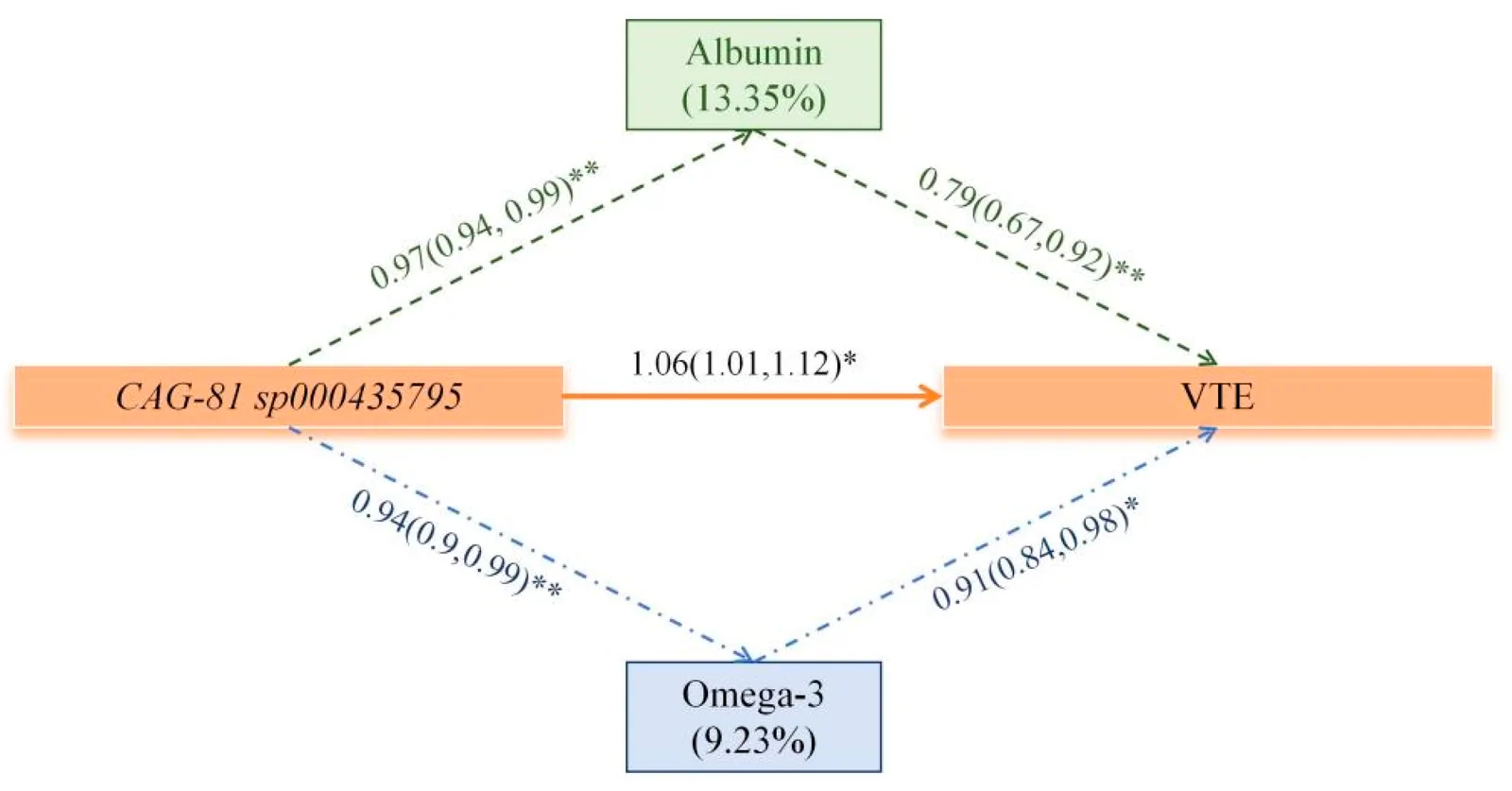
The mediated effects of the plasma metabolites. Numbers on each connecting line represent odds ratios (95% confidence interval). *: 0.01 ≤ *p* value < 0.05; **: 0.001 ≤ *p* value < 0.01; VTE: venous thromboembolism.

Considering that Omega-3 fatty acid belongs to PUFA, So we did a joint MVMR analysis including the two fatty acids. The joint MVMR results showed that PUFA was no longer associated with VTE (*p*: 0.052). Omega-3 fatty acid (*p*: 0.028) independently associated with VTE. We also evaluated the mediated effects of metabolites in gut microbiome on VTE. As shown in Figure 5, albumin, and Omega-3 fatty acid explained 13.35% and 9.23% of the association, respectively.

#### 3.2.3. Biological Function of *CAG-81 Sp000435795*

**Figure 6:**
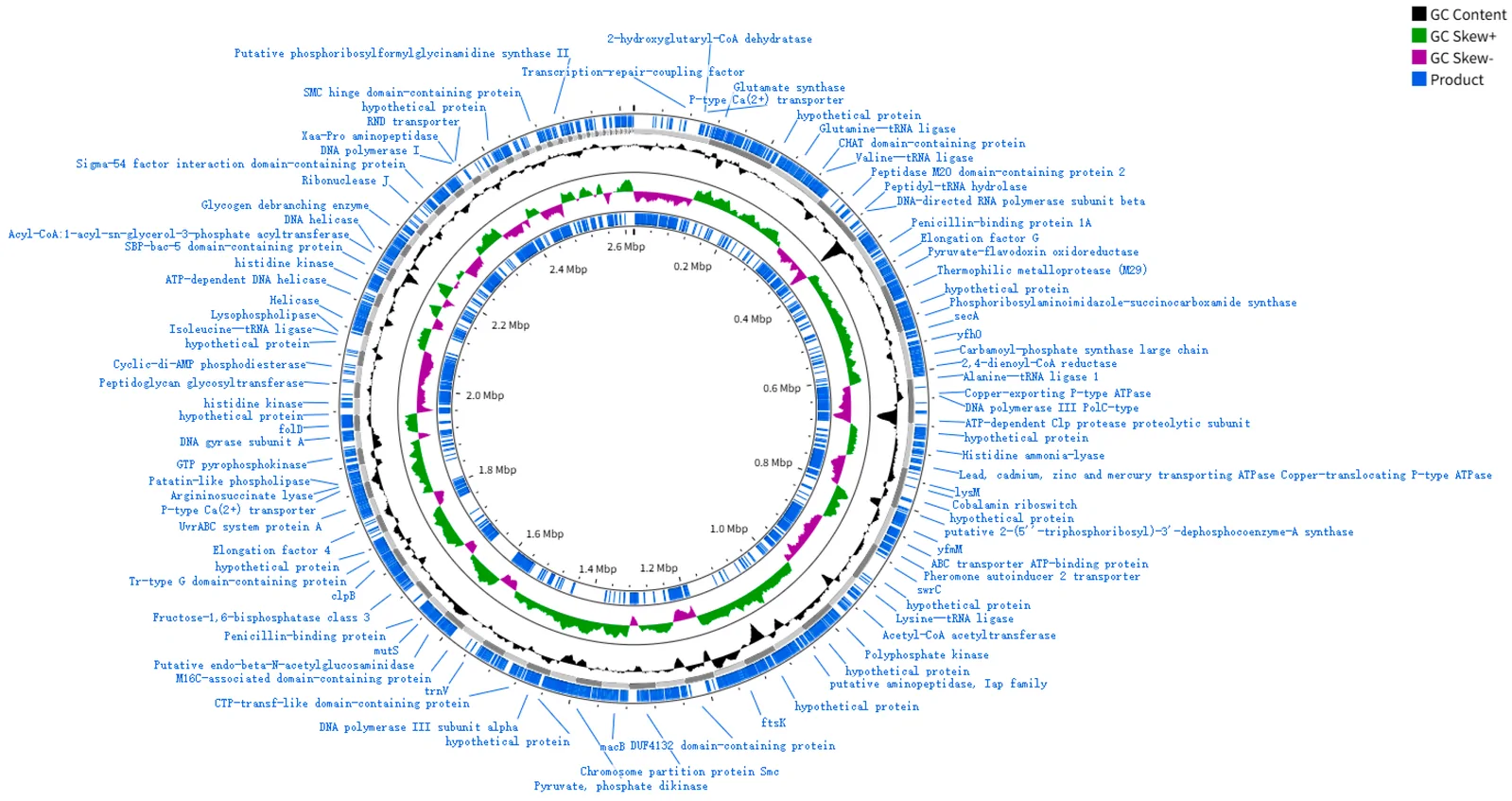
The genome annotation of *CAG-81 sp000435795*.

To explore the potential biological mechanisms underlying the observed causal relationships between *CAG-81 sp000435795* and albumin, Omega-3 levels, we performed a comprehensive functional annotation of *CAG-81 sp000435795*’s genomic context. Notably, the genomic context of *CAG-81 sp000435795* shows it might encode a set of enzymes for proteolysis, like ATP-dependent Clp protease proteolytic subunit, peptidyl-tRNA hydrolase, xaa-Pro aminopeptidase, argininosuccinate lyase, histidine ammonia-lyase, and so on. And the genomic context of *CAG-81 sp000435795* might encode a complete suite of enzymes for fatty acid degradation, such as 2,4-dienoyl-CoA reductase, acetyl-CoA acetyltransferase, acyl-CoA, lysophospholipase, and patatin-like phospholipase (as shown in Figure 6 and Supplemental Table S12).

**Figure 7:**
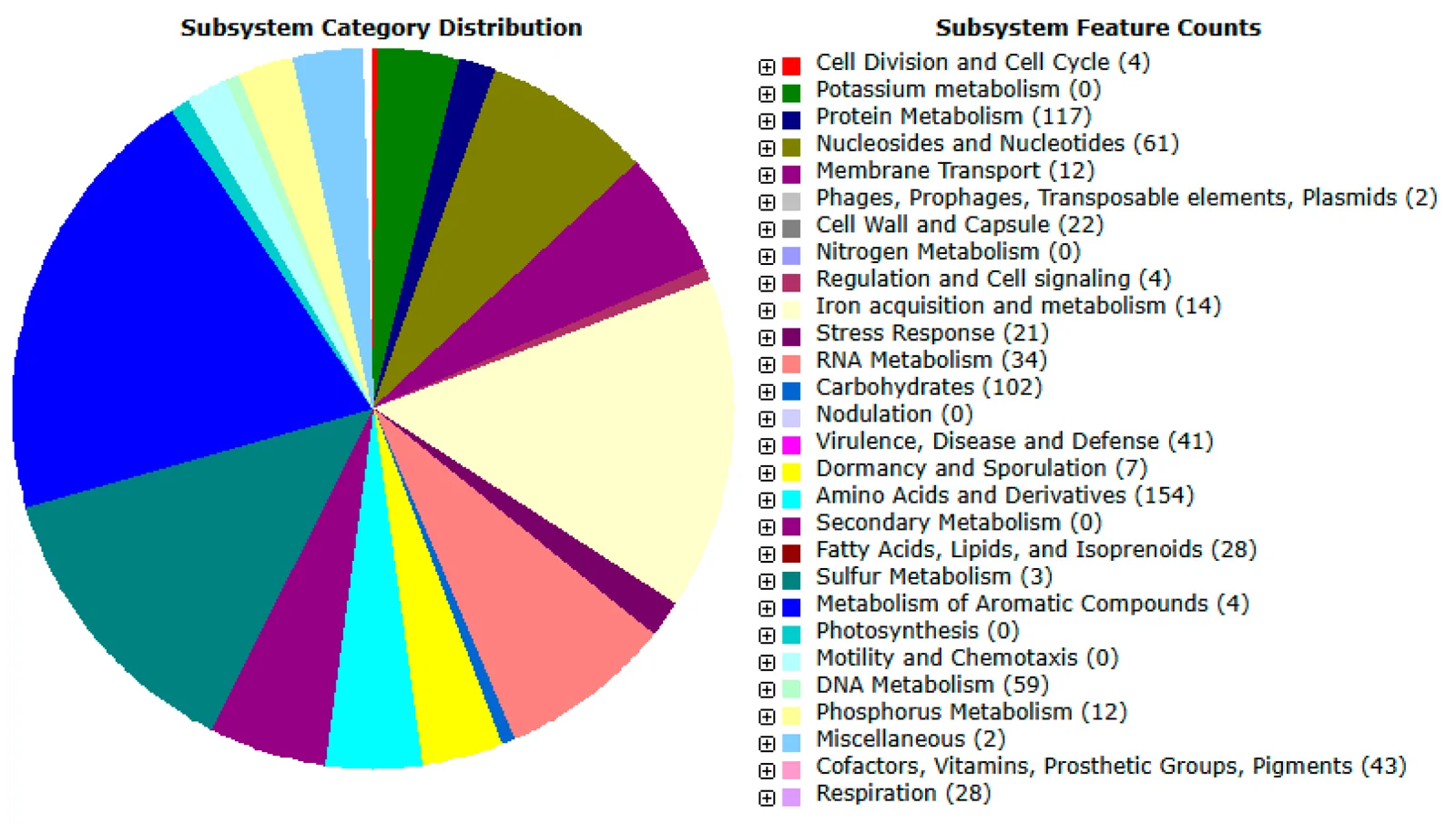
The pathway enrichment analysis of *CAG-81 sp000435795*.

As seen in Figure 7 and Supplemental Table S13, subsequent pathway enrichment analysis revealed that 117 genome products encoded by *CAG-81 sp000435795* were implicated in protein metabolism; 154 genome products might participate in amino acid and derivative metabolism; and 28 genome products might be involved in fatty acid, lipid, and isoprenoid metabolism.

## 4. Discussion

We first demonstrated the independent positive causal relationships between the two species (*CAG-81 sp000435795* and *CAG-877 sp000433455*) and VTE. Multiple sensitivity analyses confirmed the consistency with the MR analysis. And the MR result of *CAG-877 sp000433455* was based on only five instrumental SNPs, requiring replication in larger microbiota GWAS with greater instrument coverage. Furthermore, we comprehensively investigated whether the gut microbiota-associated metabolites mediated the influence of the gut microbiome on VTE. And we confirmed that *CAG-81 sp000435795* affects the risk of VTE through Omega-3 and albumin. Further functional analysis showed that *CAG-81 sp000435795* might play a role in albumin and fatty acids metabolism.

*CAG-81 sp000435795* belongs to the *Firmicutes* phylum, *Clostridia* class, *Lachnospirales* order, *Lachnospiraceae* family, *CAG-81* genus. *CAG-81 sp000435795* was found to increase the risk of VTE. To date, few studies have been carried out on the microbe. One previous study reported that *CAG-81 sp000435795* increased the risk of temporomandibular joint disorders [44]. At the level of phylum, patients with VTE have been shown to exhibit an overgrowth of *Firmicutes* [8].

*CAG-81 sp000435795* was found to decrease the level of albumin, Omega-3 fatty acid and PUFA. And through genome annotation, *CAG-81 sp000435795* was validated to encode enzymes for albumin and fatty acids degradation to regulate systemic circulating levels of albumin and fatty acids. What is more, pathway enrichment analysis has proven that *CAG-81 sp000435795* was embedded within a metabolic network involving protein and fatty acids metabolism. Several studies have proven that gut microbiota participated the synthesis and metabolism of PUFAs [45,46,47]. Chronic kidney disease associated dysbiosis is characterized by reduced short-chain fatty acid production [48]. Different compositions of gut microbiota exhibited significantly different serum albumin concentrations [49].

In the discovery and validation cohorts, albumin both causally decreased the risk of VTE. The clinical associations were consistent with our results. A comprehensive meta-analysis of more than 2 million patients has shown that patients with hypoalbuminemia had almost a two-fold risk of VTE than patients with a normal level of albumin [50]. In SARS-CoV-2 patients, serum albumin levels below 3.5 g/dL are associated with elevated D-dimer and thrombotic events [51]. Our study further supports a causal relationship between albumin and VTE. Albumin has been reported to participate in inflammatory response to antigenic stimulus, collagen metabolic process and regulation of collagen biosynthetic process, thereby contributing to protect from VTE [52]. In cerebral infarction mice, albumin administration effectively downregulated the expression of TLR4. Simultaneously, the albumin treatment promoted an anti-inflammatory environment by significantly upregulating IL-10 and TGF-β1 expression, and Tregs CD4(+) cells proportion [53], which might decrease endothelial activation [54]. TLR4 activation would increase VTE generation [55].

Long-chain Omega-3 fatty acid can be endogenously synthesized from their respective precursors via elongation and desaturation [56]. The clinical cohort studies have shown that participants with higher Omega-3 fatty acid concentrations had lower incidence of VTE and recurrent VTE [57,58]. In the present MR analysis, the Omega-3 causally decreased the risk of VTE. Emerging evidence suggests that Omega-3 fatty acids may mitigate VTE risk by attenuating inflammatory responses, downregulating adhesion molecules and improving endothelial function. Specifically, perinatal supplementation with docosahexaenoic acid (DHA), a prominent Omega-3 fatty acid, has been shown to effectively counteract the pro-inflammatory effects of maternal circadian disruption on neonatal immune responses [59]. Mechanistically, Omega-3 fatty acids regulate the expression of inflammation-related genes by modulating key transcription factors, including nuclear factor-κB (NF-κB) and peroxisome proliferator-activated receptor-γ (PPAR-γ) [60,61]. Furthermore, Omega-3 fatty acids downregulate the expression of critical adhesion molecules [62], such as vascular cell adhesion molecule-1 (VCAM-1) and intercellular adhesion molecule-1 (ICAM-1) [63,64], which are essential for the recruitment of inflammatory cells to sites of inflammation. Beyond these anti-inflammatory effects, Omega-3 fatty acids have been reported to improve vascular endothelial function, facilitating the recovery of vasoconstrictive and vasodilatory capacities during sepsis [65].

In the present study, we did not confirm the causal relationship between LPS and VTE. LPS has been shown to accelerate clot formation in previous experimental settings [66]. As a component of the outer membrane of all Gram-negative bacteria, LPS is primarily released upon bacterial cell death or during the formation of outer membrane vesicles [67]. The limited capacity of host hereditary gene variants to capture the sudden bacterial cell death might partly explain the discrepancy between the results of the previous study and our current MR-based findings. And the limited IVs of LPS in the study may cause false negative results.

Utilizing summary statistics from the largest GWAS of VTE, this study systematically investigated the causal relationships between specific gut bacterial species and VTE. Furthermore, the mediating effects of gut microbiota-related metabolites were evaluated. However, the study has several limitations. Firstly, the sample size of the gut microbiome cohort is 5,959; due to the sample size discrepancies between the exposure and outcome cohorts, the mediated effect might be affected. Furthermore, the types of species remain to be explored. As the number of species databases increases, it is expected that additional species implicated in this pathological process will be identified and characterized. Secondly, the underlying biological mechanisms in the study were based on MR analysis and functional analysis. They still warrant further elucidation through in vivo and in vitro experimentation. In light of the fact that the data included in this study were derived primarily from individuals of European ancestry, the generalizability of the findings to other ethnic or geographic populations may be limited.

## 5. Conclusions

Utilizing the largest available GWAS summary data, we conducted mediated Mendelian randomization analysis to investigate the roles of gut-related metabolites and endotoxins in mediating the effect of the specific gut species on venous thrombosis. *CAG-81 sp000435795* causally increased the risk of VTE by decreasing the levels of metabolites (albumin, Omega-3 fatty acid). Albumin and Omega-3 fatty acid might be safe markers for VTE risk stratification and therapeutic intervention.

## Figures and Tables

**Table 1:** Results of multivariate Mendelian Randomization analysis.

Species	Outcome	Beta	Se	OR (95%CI)	*p*
*CAG-776 sp000438195*	VTE	0.03	0.04	1.03 (0.95, 1.11)	0.44
*CAG-81 sp000435795*	VTE	0.06	0.02	1.06 (1.02, 1.1)	0.004
*CAG-877 sp000433455*	VTE	0.12	0.05	1.13 (1.02, 1.24)	0.03

VTE: venous thromboembolism.

## Data Availability

VTE GWAS data could be downloaded from https://www.decode.com/summarydata (accessed on 31 Augst 2023). Summary data for gut microbiota and plasma metabolites could be obtained from the MRC Integrative Epidemiology Unit (IEU) open GWAS project (https://openGWAS.io/datasets/ (accessed on 19 May 2025)). And replication cohorts for PUFA and the Omega-3 were obtained from the GWAS Catalog database (https://www.ebi.ac.uk/gwas/ (accessed on 6 June 2025)).

## Supplementary Materials

The following supporting information can be downloaded at: https://www.mdpi.com/article/10.3390/genes17091098/s1, Supplemental Figure S1. A. Leave-one-out test plot; B. scatter plot; C. funnel plot of the *CAG-81 sp000435795* on VTE risk. Supplemental Figure S2. A. Leave-one-out test plot; B. scatter plot; C.funnel plot of the causal effect of *CAG-776 sp000438195* on VTE risk. Supplemental Figure S3. A. Leave-one-out test plot; B. scatter plot; C. funnel plot of the causal effect of *Holdemania sp900120005* on VTE risk. Supplemental Figure S4. A. Leave-one-out test plot; B. scatter plot; C. funnel plot of the causal effect of *CAG-877 sp000433455* on VTE risk. Supplemental Figure S5. A. Leave-one-out test plot; B. scatter plot; C. funnel plot of the causal effect of *CAG-180 sp000432435* on VTE risk. Supplemental Figure S6. A. Leave-one-out test plot; B. scatter plot; C. funnel plot of the causal effect of *F. lactaris* on VTE risk. Supplemental Figure S7. A. Leave-one-out test plot; B. scatter plot; C. funnel plot of the causal effect of *CAG-81 sp000435795* on albumin. Supplemental Figure S8. A. Leave-one-out test plot; B. scatter plot; C. funnel plot of the causal effect of *CAG-81 sp000435795* on Omega-3. Supplemental Figure S9. A. Leave-one-out test plot; B. scatter plot; C. funnel plot of the causal effect of *CAG-81 sp000435795* on PUFA. Supplemental Figure S10. A. Leave-one-out test plot; B. scatter plot; C. funnel plot of the causal effect of albumin on VTE risk. Supplemental Figure S11. A. Leave-one-out test plot; B. scatter plot; C. funnel plot of the causal effect of Omega-3 on VTE risk. Supplemental Figure S12. A. Leave-one-out test plot; B. scatter plot; C. funnel plot of the causal effect of PUFA on VTE risk. Supplemental Table S1: Detailed information of including datasets; Supplemental Table S2: STROBE-MR Checklist; Supplemental Table S3: Instrumental variables selection of suggestive genome-wide significant gut microbiome loci; Supplemental Table S4: Instrumental variables selection of genome-wide significant VTE loci; Supplemental Table S5: Instrumental variables selection of genome-wide significant lipids loci; Supplemental Table S6: Mendelian Randomization results between species of gut microbiome and VTE; Supplemental Table S7: Mendelian Randomization results between metabolites and VTE; Supplemental Table S8: Mendelian Randomization results between Gut Microbiome indepently significantly associated with VTE and metabolites; Supplemental Table S9: MR PRESSO test results on IVs of significant MR associations; Supplemental Table S10: Steiger test results for significant MR analysis Results; Supplemental Table S11: Reverse MR analysis Results; Supplemental Table S12: Genome annotation of *CAG-81 sp000435795*; Supplemental Table S13: The pathway enrichment analysis of *CAG-81 sp000435795*.
