# Supplementary material for: Genetic Evidence for Metabolites Mediated Causal Inference Between Gut Microbiota and Venous Thromboembolism
Source: Genes (Basel). 2026 Sep 11;17(9):1098. doi: 10.3390/genes17091098 (PMC13606309; doi:10.3390/genes17091098)
Supplement: Supplementary file 1 [file genes-17-01098-s001.zip › Supplemental Figures.pdf]

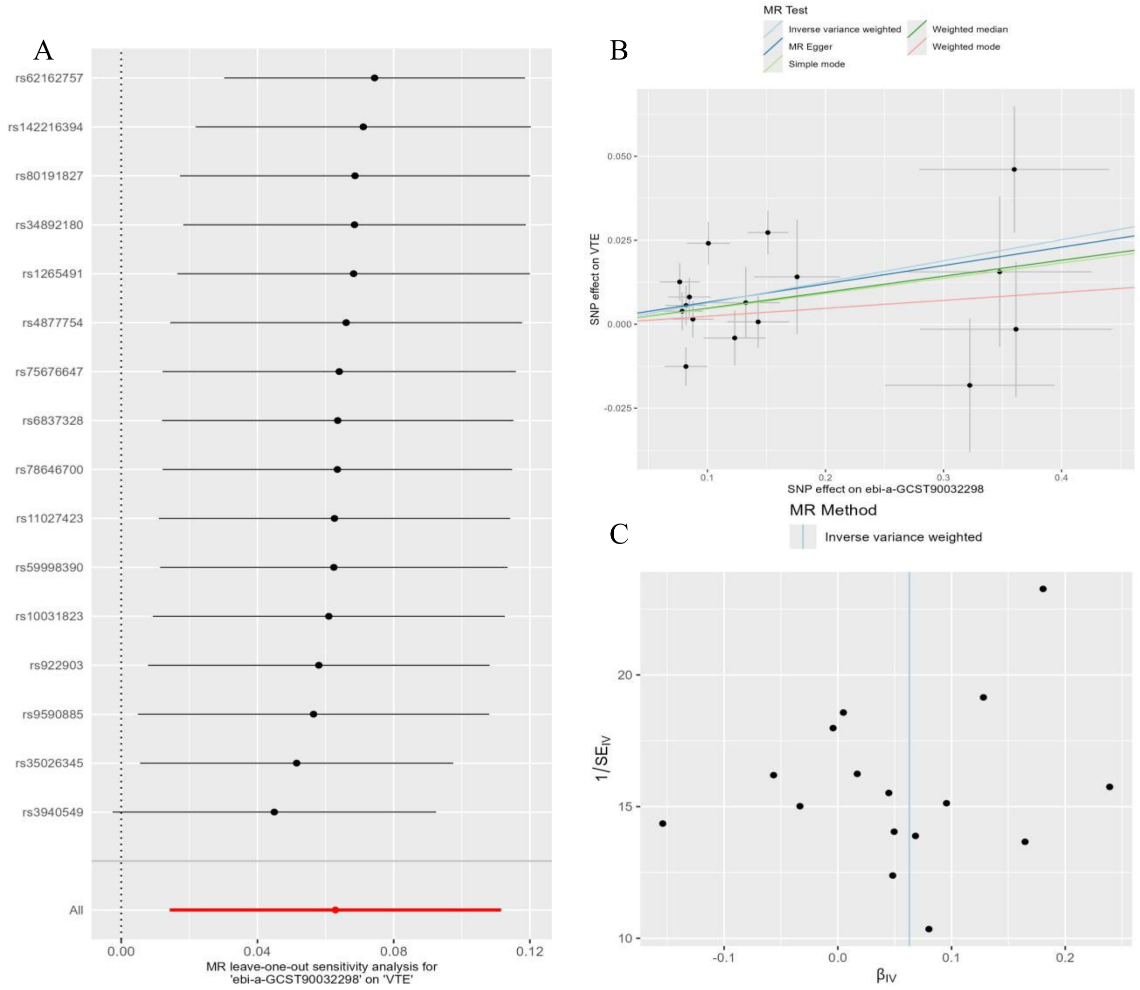

Supplemental Figure S1. A. Leave-one-out test plot; B. scatter plot; C. funnel plot of the *CAG-81 sp000435795* on VTE risk.

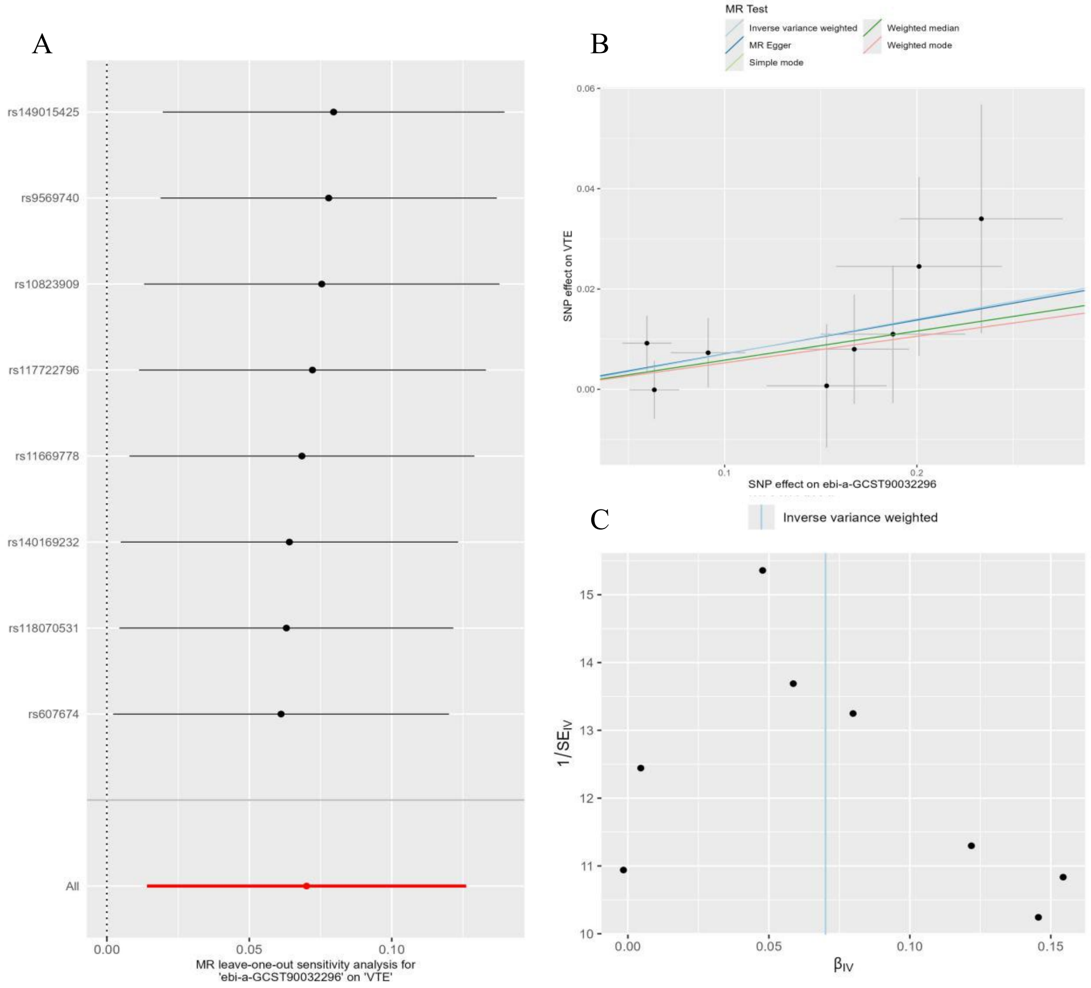

Supplemental Figure S2. A. Leave-one-out test plot; B. scatter plot; C.funnel plot of the causal effect of *CAG-776 sp000438195* on VTE risk.

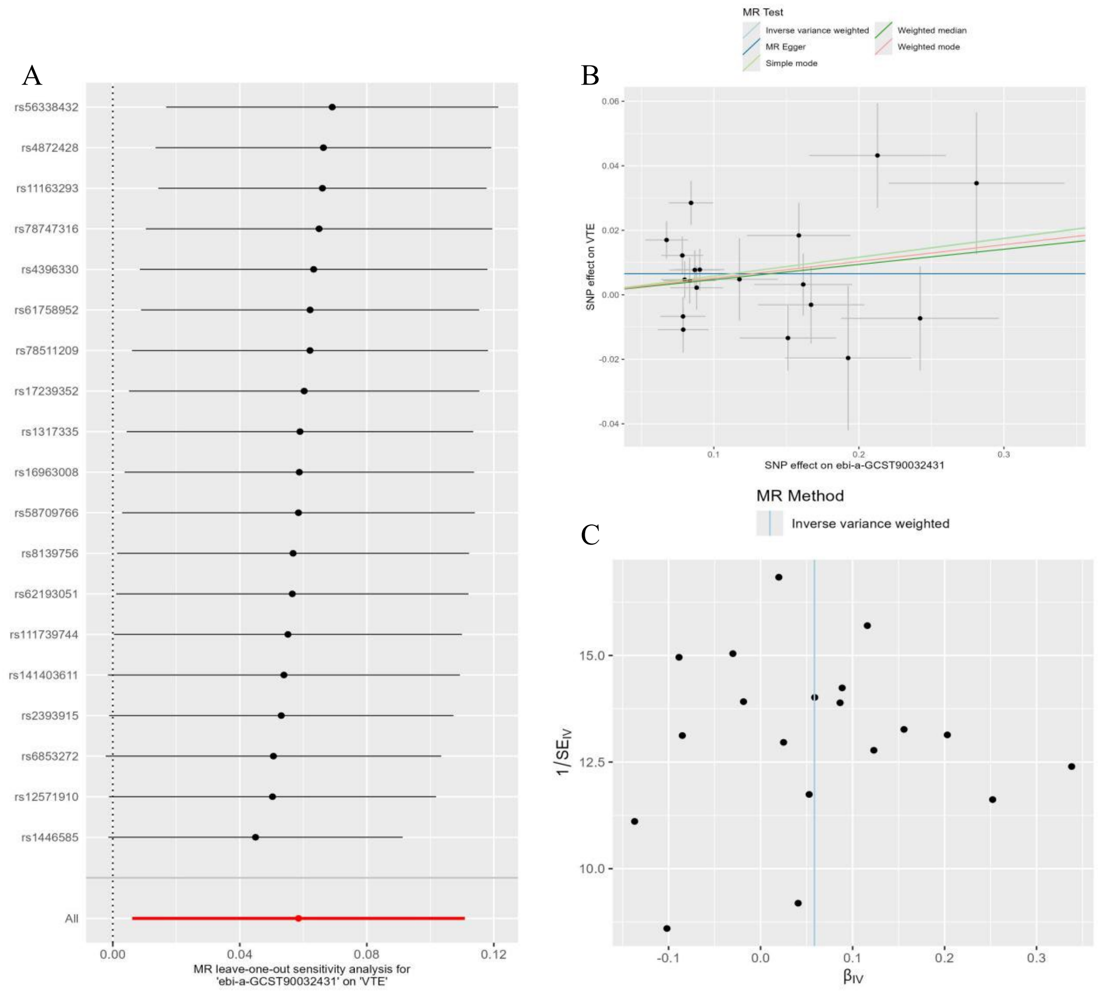

Supplemental Figure S3. A. Leave-one-out test plot; B. scatter plot; C. funnel plot of the causal effect of *Holdemania sp900120005* on VTE risk.

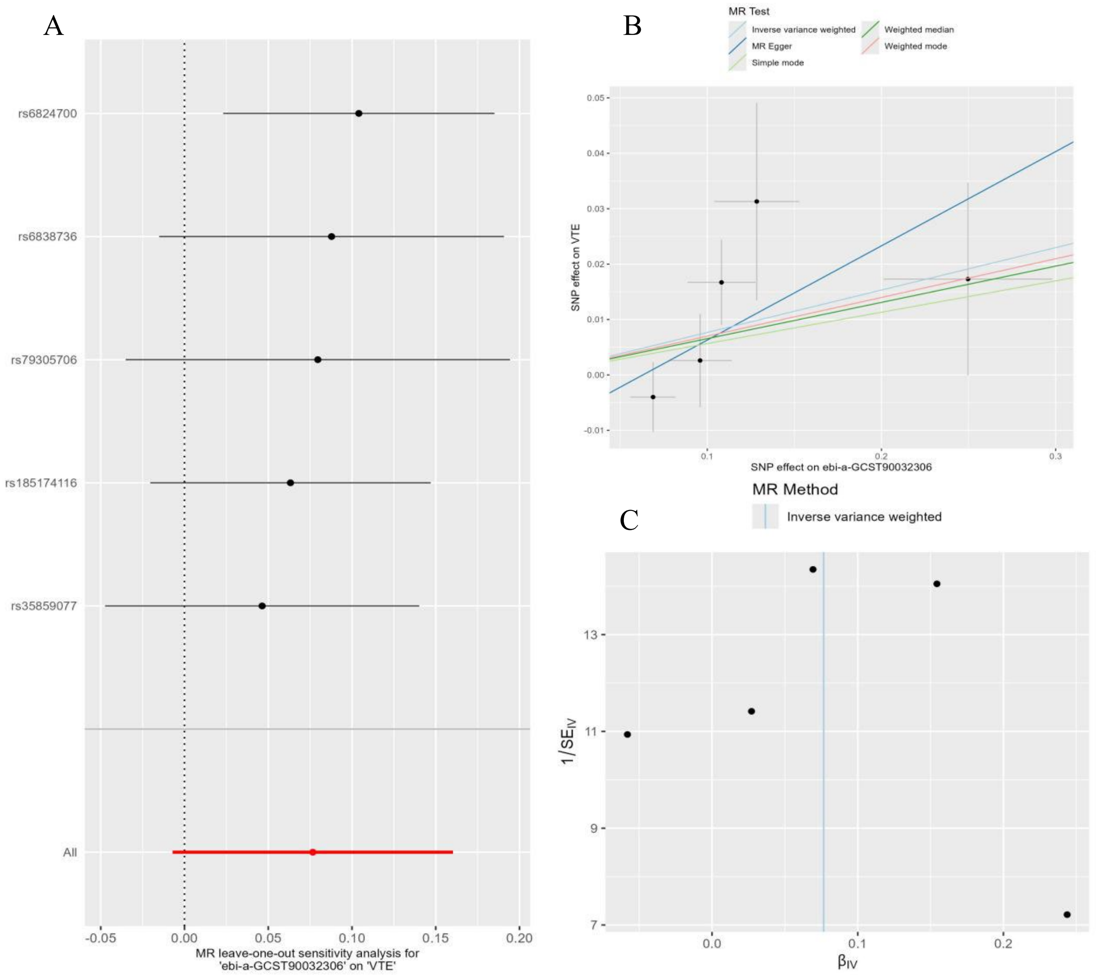

Supplemental Figure S4. A. Leave-one-out test plot; B. scatter plot; C. funnel plot of the causal effect of *CAG-877 sp000433455* on VTE risk.

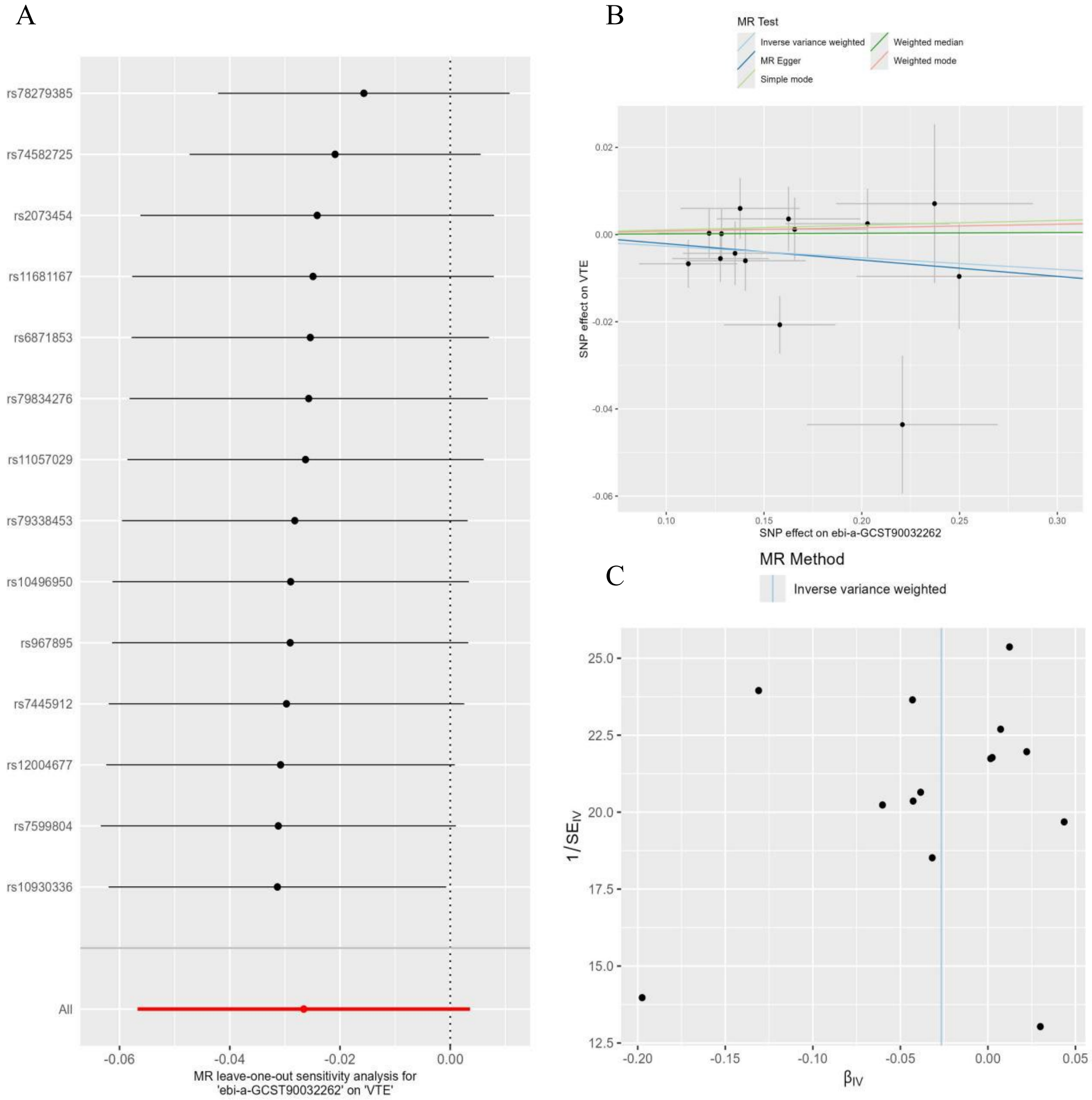

Supplemental Figure S5. A. Leave-one-out test plot; B. scatter plot; C. funnel plot of the causal effect of *CAG-180 sp000432435* on VTE risk.

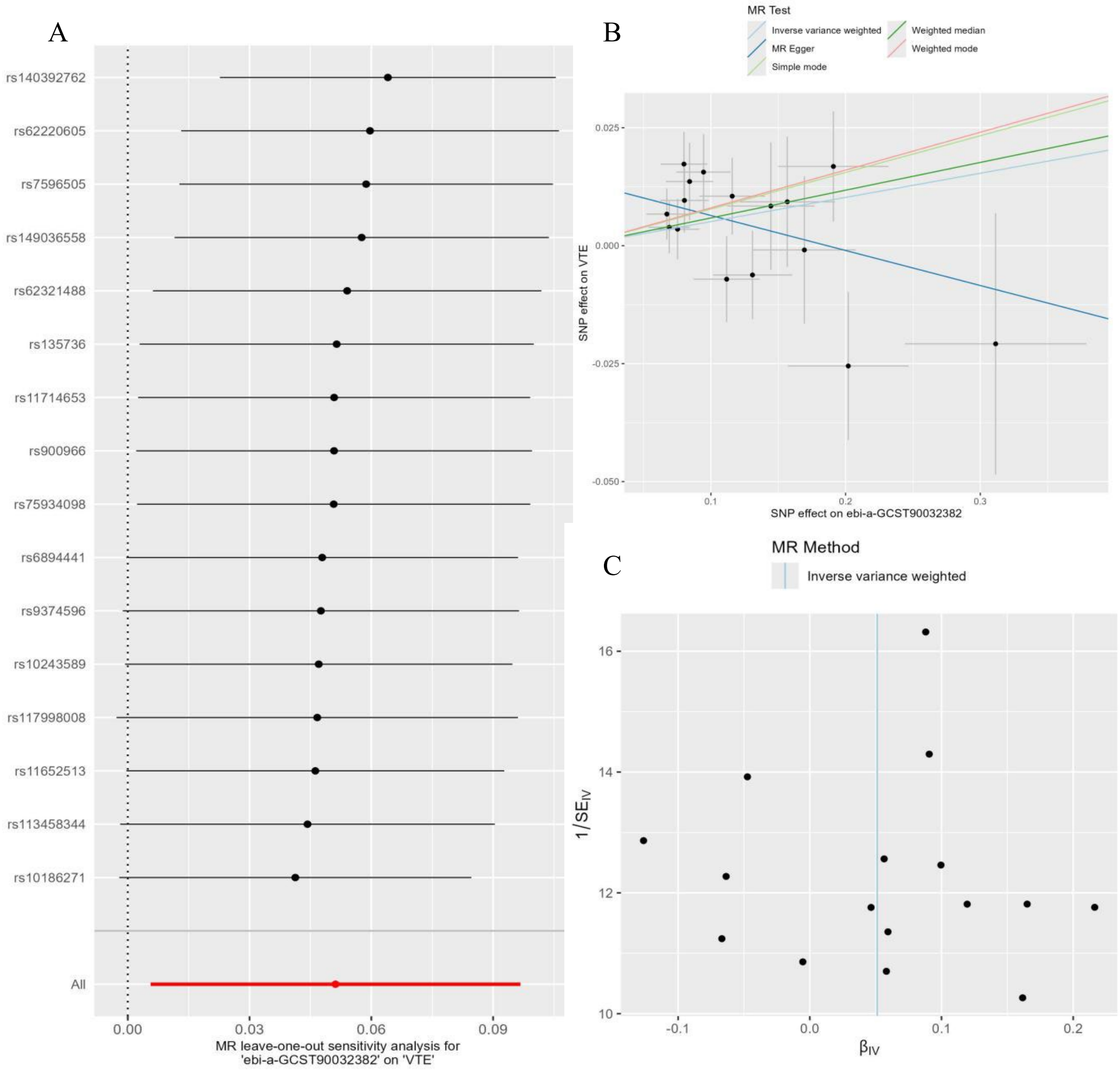

Supplemental Figure S6. A. Leave-one-out test plot; B. scatter plot; C. funnel plot of the causal effect of *Faecalicatena lactaris* on VTE risk.

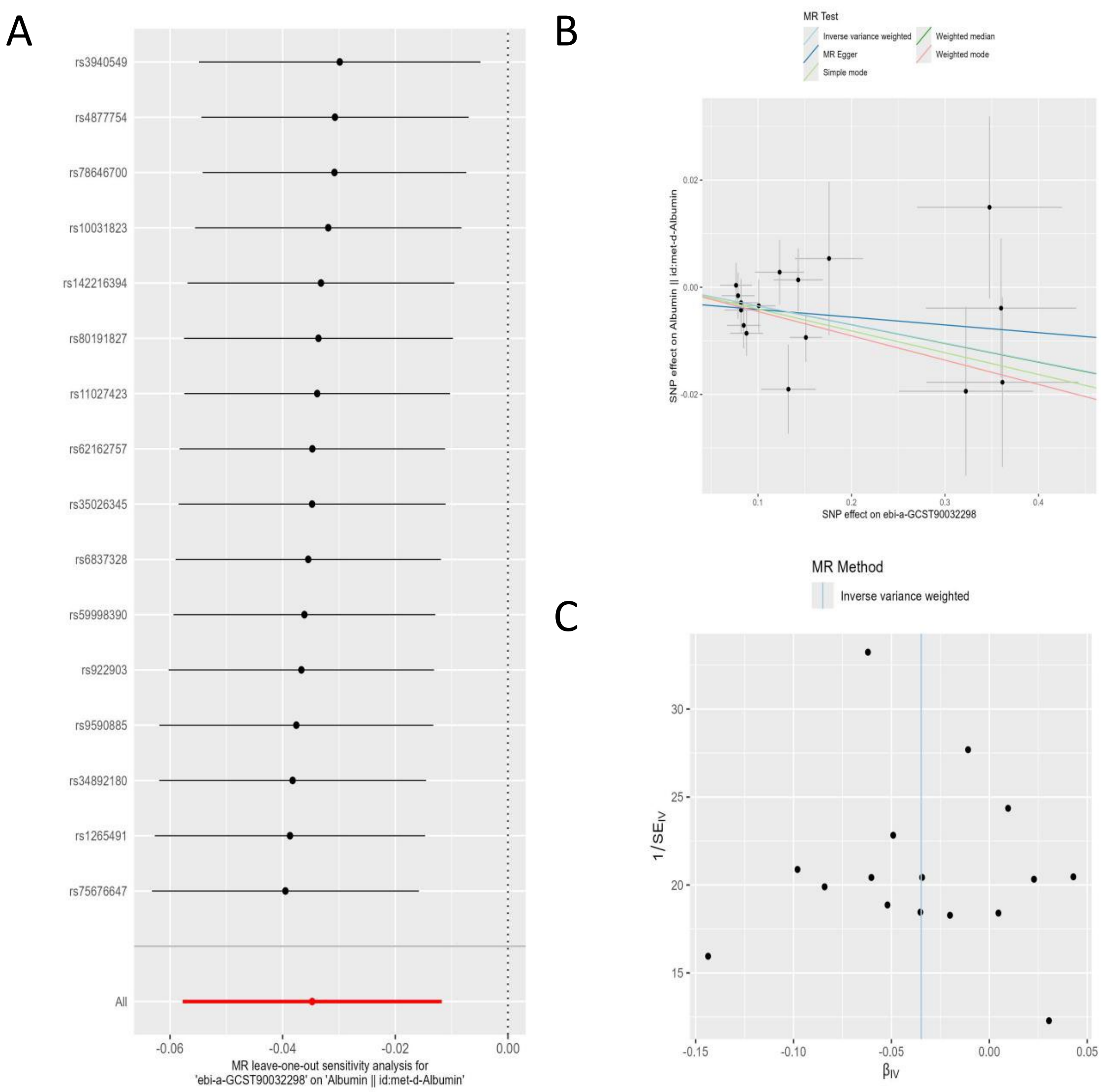

Supplemental Figure S7. A. Leave-one-out test plot; B. scatter plot; C. funnel plot of the causal effect of *CAG-81 sp000435795* on albumin.

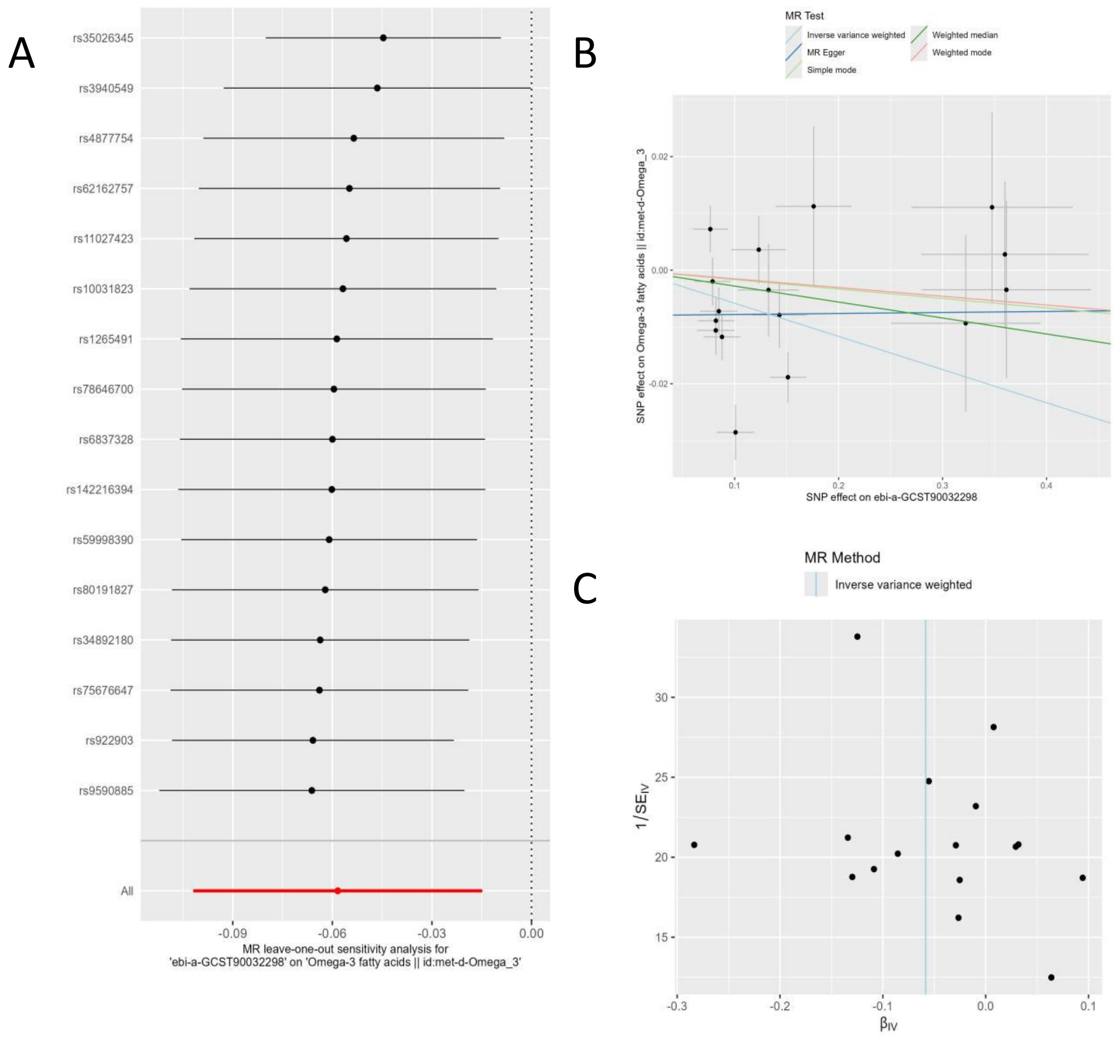

Supplemental Figure S8. A. Leave-one-out test plot; B. scatter plot; C. funnel plot of the causal effect of *CAG-81 sp000435795* on Omega-3.

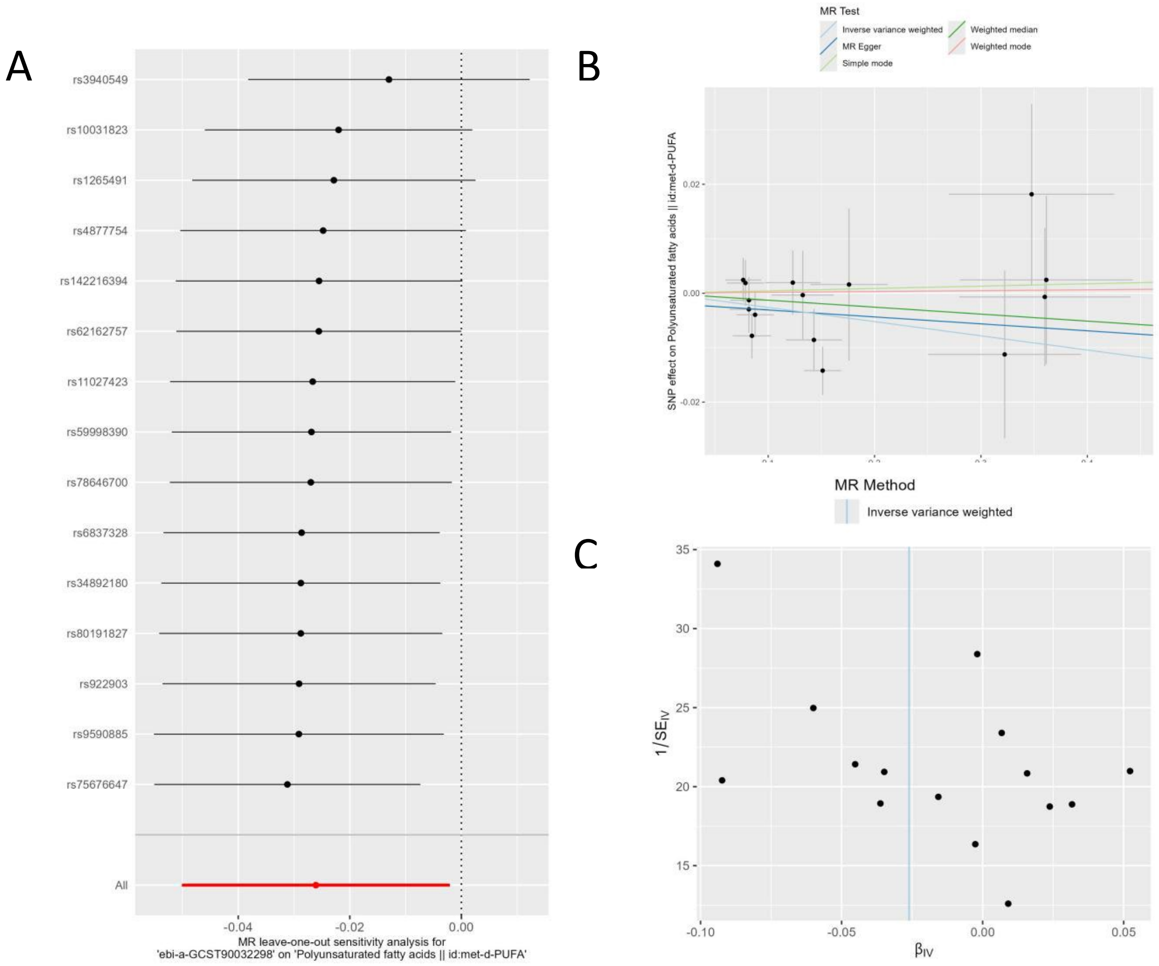

Supplementalal Figure S9. A. Leave-one-out test plot; B. scatter plot; C. funnel plot of the causal effect of *CAG-81 sp000435795* on PUFA.

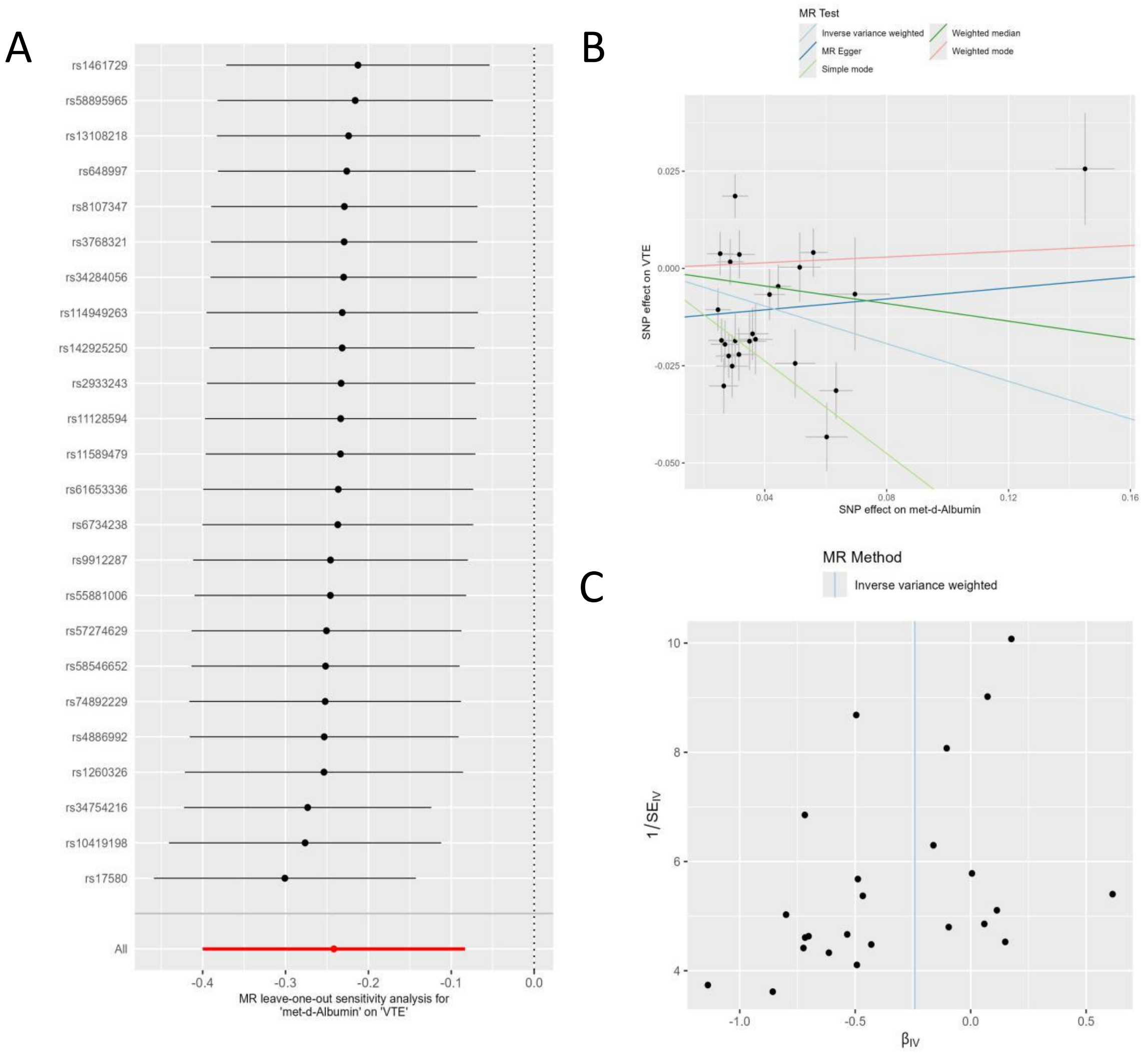

Supplemental Figure S10. A. Leave-one-out test plot; B. scatter plot; C. funnel plot of the causal effect of albumin on VTE risk.

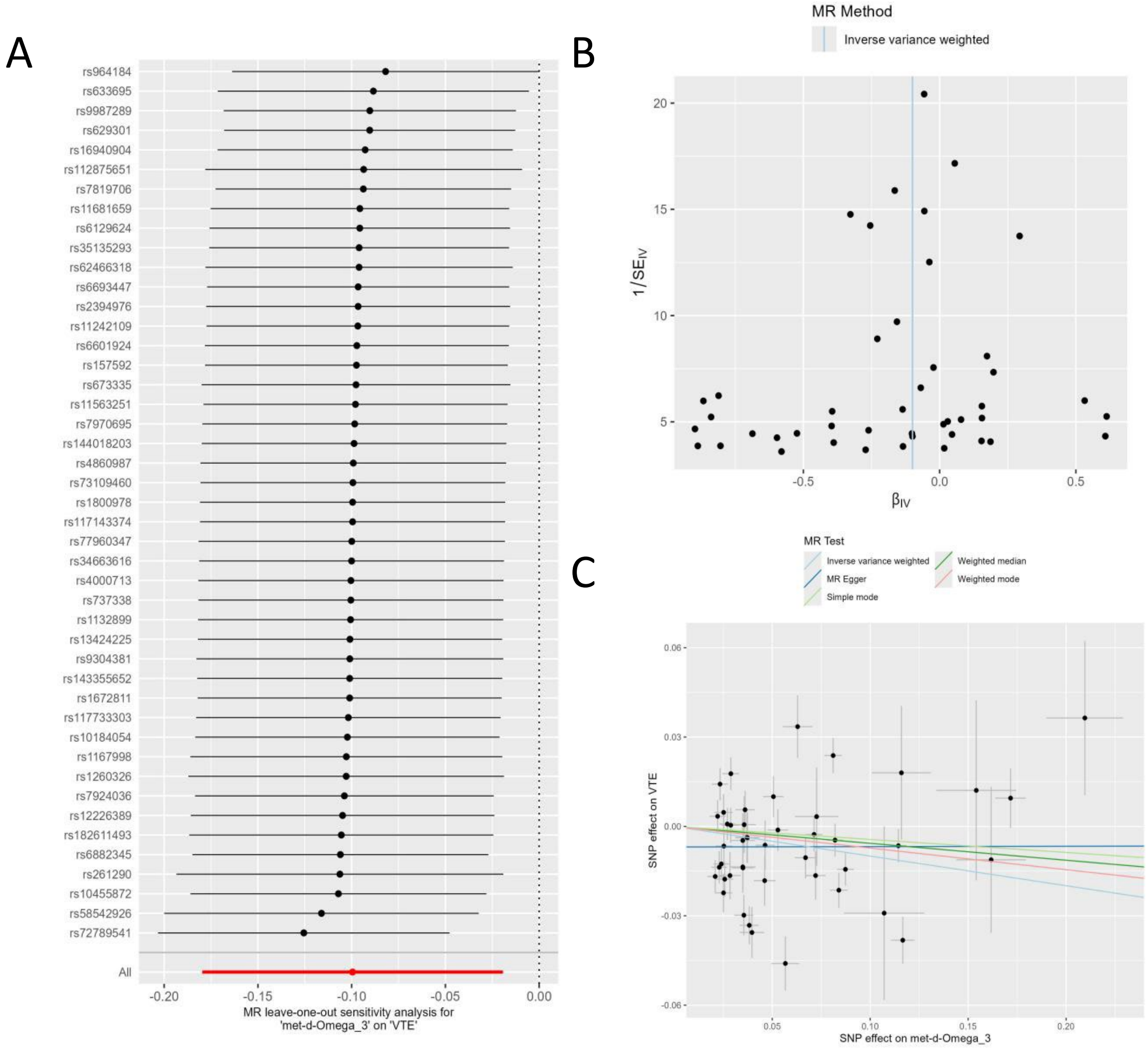

Supplemental Figure S11. A. Leave-one-out test plot; B. scatter plot; C. funnel plot of the causal effect of Omega-3 on VTE risk.

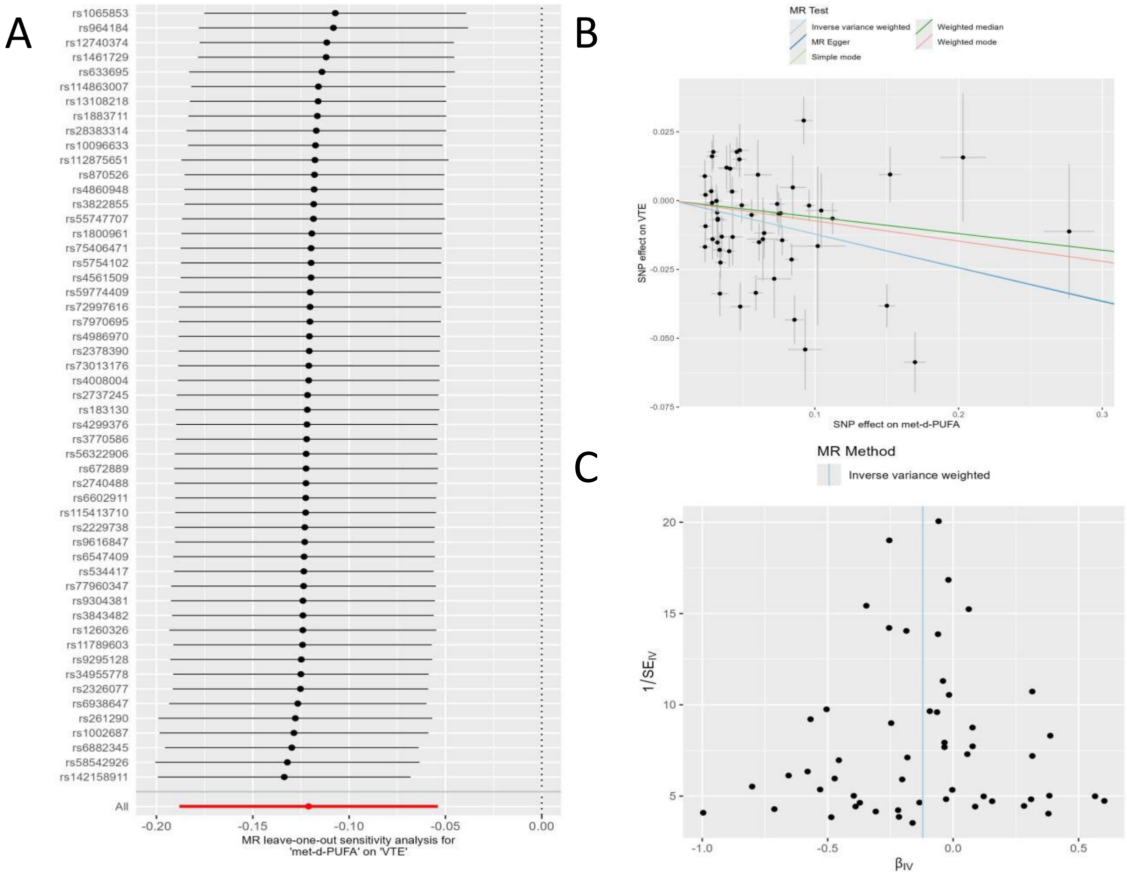

Supplemental Figure S12. A. Leave-one-out test plot; B. scatter plot; C. funnel plot of the causal effect of PUFA on VTE risk.
